# Leveraging Edge Computing for Video Data Streaming in UAV-Based Emergency Response Systems

**DOI:** 10.3390/s24155076

**Published:** 2024-08-05

**Authors:** Mekhla Sarkar, Prasan Kumar Sahoo

**Affiliations:** 1 Department of Computer Science and Information Engineering, Chang Gung University, Guishan, Taoyuan 33302, Taiwan; d0829005@cgu.edu.tw; 2 Department of Neurology, Chang Gung Memorial Hospital, Linkou Medical Center, Guishan, Taoyuan 333423, Taiwan

**Keywords:** unmanned aerial vehicle (UAV), edge computing, resource management, video data stream, bandwidth allocation

## Abstract

The rapid advancement of technology has greatly expanded the capabilities of unmanned aerial vehicles (UAVs) in wireless communication and edge computing domains. The primary objective of UAVs is the seamless transfer of video data streams to emergency responders. However, live video data streaming is inherently latency dependent, wherein the value of the video frames diminishes with any delay in the stream. This becomes particularly critical during emergencies, where live video streaming provides vital information about the current conditions. Edge computing seeks to address this latency issue in live video streaming by bringing computing resources closer to users. Nonetheless, the mobile nature of UAVs necessitates additional trajectory supervision alongside the management of computation and networking resources. Consequently, efficient system optimization is required to maximize the overall effectiveness of the collaborative system with limited UAV resources. This study explores a scenario where multiple UAVs collaborate with end users and edge servers to establish an emergency response system. The proposed idea takes a comprehensive approach by considering the entire emergency response system from the incident site to video distribution at the user level. It includes an adaptive resource management strategy, leveraging deep reinforcement learning by simultaneously addressing video streaming latency, UAV and user mobility factors, and varied bandwidth resources.

## 1. Introduction

In recent years, the global community has witnessed numerous catastrophic events, such as the Tohoku earthquake and tsunami in Japan, the Fani cyclone in India [1], or fire conditions in deep forests [2]. These disasters have resulted in the widespread devastation of infrastructure and significant loss of human life. The initial hours following such events are crucial for providing urgent assistance and potentially saving many lives. However, the aftermath of a disaster often involves the collapse of the existing infrastructure and communication systems, leaving affected areas isolated and without means of exchanging vital information. In such a dynamic and challenging environment, establishing an effective emergency communication network holds significant importance for facilitating emergency rescue operations, particularly in scenarios where the communication infrastructure, such as ground base stations (GBSs), is subjected to damage or located at a distance.

The incorporation of Internet of Things (IoT) technologies, specifically unmanned aerial vehicles (UAVs), presents a promising opportunity to enhance efficiency and effectiveness, and minimize the exposure of response personnel to hazardous environments. The higher mobility and adaptability of UAVs together with advanced transceivers can support essential communication, which can be utilized as real-time data collectors or flying base stations [3,4,5]. IoT-enabled UAVs present a versatile platform by gathering critical information as well as providing fast, seamless, and reliable cellular communication [6]. However, in general, the affected areas lie far from the working ground base station (GBS). So to retrieve information from the affected area in the form of video transmission, many recent works [2,7,8,9] have focused on the inclusion of a UAV-aided relaying system. This relaying UAV, also known as Link UAV (LUAV), facilitates data transmission between ground-based devices such as GBS and other monitoring UAVs (MUAV) by relaying signals, providing on-demand communication services to those areas without network coverage. The study in [10,11] has shown considerable potential in integrating mobile edge computing (MEC) with UAVs. For instance, Ref. [10] used MEC in addressing the computing needs of maritime terminals facing resource scarcity or latency sensitivity. This approach is tailored to meet the demands of maritime users, offering advantages such as big data support, low latency, cost effectiveness, and high reliability. Unlike remote cloud centers [12], MEC servers boast robust computing and storage capabilities while being situated close to the network edge. This proximity minimizes transmission latency and energy consumption. MEC further expands its applicability to the Internet of Vehicles (IoV) as demonstrated in a study by [4,13,14].

The study in [4] concentrated on controlling the bandwidth trajectory of UAVs to optimize system communication capacity, facilitating the efficient processing of IoV-related data. Ref. [13] integrated task sequencing and resource allocation to effectively handle computing and communication resources simultaneously. Additionally, Ref. [14] aimed to maximize network spectrum-energy efficiency. However, the data collected by UAVs, particularly video data, typically display strong inter-frame dependencies along with high temporal and spatial dimensions. This necessitates specific codec operations, including compression and decompression, such as H.264, H.265 (HEVC), VP9, and AV1 [15], each of which requires varying computational resources. The transmission and storage of video data entail significant bandwidth requirements due to their large size and variable bit rate characteristics. Additionally, the velocity of the UAV and its trajectory play crucial roles in determining the real-time transmission and processing capabilities of video data, as higher velocities may introduce challenges in maintaining stable communication links and processing video streams efficiently during flight. To solve these challenges, UAV technology can be integrated with MEC networks. Specifically, MUAV can collect data and transfer them to LUAV for further processing. Nevertheless, due to their size, UAVs, in general, have limited computational resources, as well as communication resources.

The limited resources can become a bottleneck if both video processing and communications resource distribution are done by the LUAV, particularly in situations that demand a quick response. To efficiently handle the situation, coordination with the edge server at the GBS offers a promising and optimal solution. Edge servers at the GBS can act as central hubs, and play a pivotal role in supplying bandwidth to both relay UAVs and end users. The edge server has computational and communication resources that are better than those of UAVs, although they may not be as extensive as those found in cloud centers. The base edge server’s involvement is essential in dynamically allocating resources, managing network traffic, and ensuring seamless communication between link UAVs and end users. This coordination is vital for maintaining stable communication links, optimizing bandwidth utilization, and supporting efficient video streaming in UAV networks. Although the edge server at the GBS has sufficient communication resources, the demand for mobile video streaming is increasing, both in everyday life and in rescue operations, and has become a dominant factor in global mobile data traffic. A report by Cisco highlights that consumer internet video traffic will reach 240.2 EB by 2022 [16], while the Ericsson Mobility Report for 2022 [17] shows that video streaming constitutes a significant and rapidly expanding portion of mobile data traffic. In 2022, video traffic accounted for 70% of all cellular data, with projections indicating a rise to 80% in the coming years.

**Figure 1:**
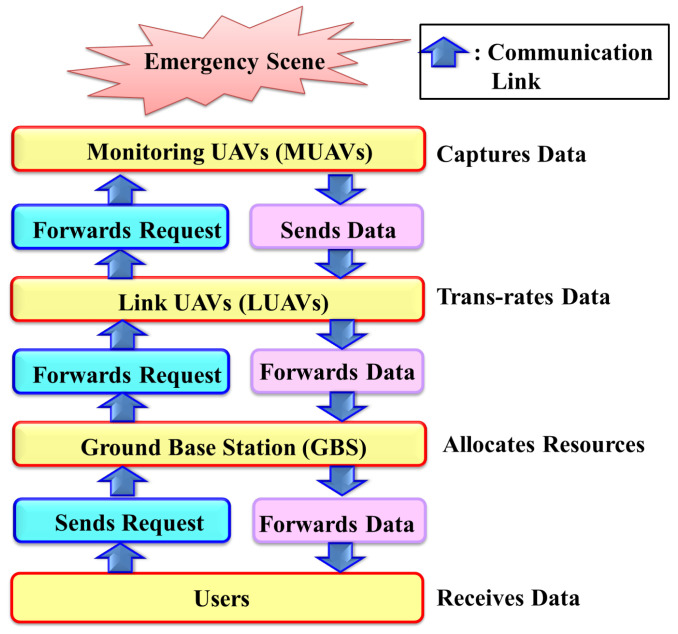
Emergency response systems.

The increasing surge in video streaming popularity has led to congestion in the network, which results particularly for users located at the edge of cellular coverage, resulting in a degradation of the quality of experience (QoE) for these end users. As a result, the base edge server has to distribute its communication resources efficiently. Recent studies in UAV technology such as [1] have explored the use of UAVs in designing emergency communication networks, utilizing Wi-Fi access points as the communication medium. The authors in [3] jointly optimized UAV trajectory and scheduling to provide wireless service to ground devices with surviving base stations. Ref. [2], on the other hand, focused on the utility-oriented optimization of UAV power, video transcoding policy, flight trajectory, and computational resource allocation using deep reinforcement learning (DRL). They ignored how the base server responds to the end users. Ref. [18] concentrated on developing intelligent UAV trajectory planning to enable energy-efficient and secure data collection. Meanwhile, Ref. [9] addressed the video resolution, movement, and power control of UAVs to maximize the QoE of real-time video streaming. We believe the additional resource management at the base server will improve the end user’s QoE. Therefore, this paper considers a physical scenario where IoT-enabled MUAVs, LUAVs, edge servers, and users collaborate to manage emergencies. The general workflow is depicted in Figure 1.

The main contributions of our work can be summarized as follows:

1. This study was conducted in a collaborative multi-user, multi-UAV scenario to handle transmission latency among collaborative users and UAVs. Emphasis was placed on the overall QoE of the system by maintaining minimum standard deviations in the transmission time among collaborative users and UAVs by jointly optimizing bandwidth allocation, video transmission, UAV trajectory control, and collaborative decision-making.
2. The softmax-aided Deep Deterministic Policy Gradient (DDPG) algorithm using deep reinforcement learning (DRL) was utilized to minimize the total transmission time among collaborative users.
3. The complex challenges associated with real-time communication and resource allocation during emergencies were addressed by considering the entire transmission process, including transmission delays from the relay UAV to the edge server, processing delays at the edge server, and transmission delays from the edge server to users.

The rest of the paper is organized as follows. The proposed system model is described in Section 2, and the problem formulation is given in Section 3. The DRL-based model is developed in Section 4. Performance evaluation of the proposed models is conducted in Section 5, and concluding remarks are made in Section 6.

## 2. System Model

**Figure 2:**
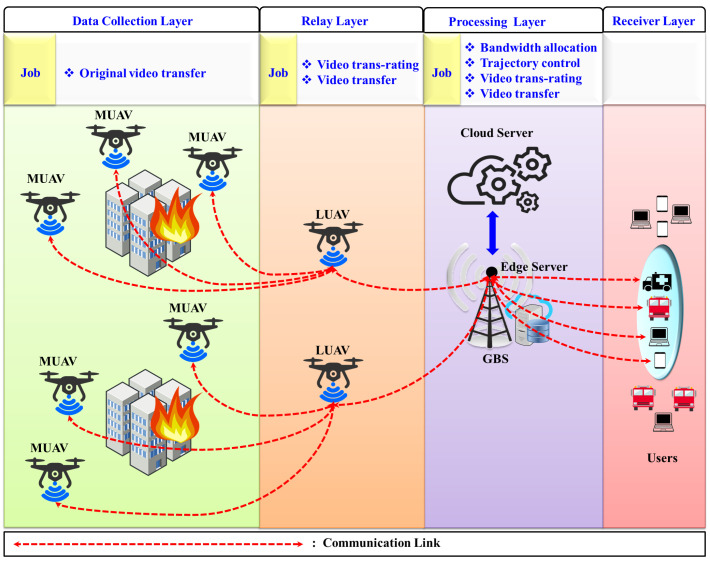
UAV-assisted edge computing scenario for emergency response systems.

In the considered scenario as shown in Figure 2, it is assumed that MUAVs have been deployed at the site of emergencies, which relays its collected data to the IoT-enabled LUAV with MEC. LUAV then forwards those data to the base edge servers. The base edge server provides communication resources for both LUAV and end users. In addition to its other data-processing functions, the LUAV is utilized as the primary video data trans-rating mechanism. If the available bandwidth fails to meet the permissible delay of the users, the edge server will engage in further trans-rating of the received data. The users being referred to in this context are emergency rescuers who operate collaboratively. To enhance the QoE of end users, DRL emerges as a promising approach. DRL algorithms have proven to be particularly adept at complex decision-making tasks and adapting to dynamic environments as can be seen in managing resources in complex cloud centers [19], making them an ideal choice for optimizing resource allocation in UAV networks. By leveraging experiential learning and environmental feedback, DRL-based solutions can continuously update resource allocation strategies, resulting in superior performance in comparison to the traditional approach, which requires prior knowledge of the environment.

As shown in Figure 2, we define the set of collaborative users as U={1,2,…,U}, which submits the request for live video streaming of the emergency area to the nearest $g^{th}$ edge server at GBS. Let GBS be defined as G={1,2,3,…,G} with spatial location of $p_{t}^{g}=\left(x_{t}^{g},y_{t}^{g},0\right)\in R^{3X1}\forall g\in G$. These users work in collaboration. We define the locations of these users as $p_{t}^{u}=\left(x_{t}^{u},y_{t}^{u},0\right)\in R^{3X1}\forall u\in U$, having a spatial velocity of $v_{t}^{u}$.

In general, UAVs possess limited communication range, rendering them unsuitable for establishing direct communication links with the edge server or users located far away from it. Consequently, a relay system involving intermediate link UAVs is often employed to facilitate data transmission between the MUAVs and the edge server or users, ensuring effective communication despite the inherent limitations of the UAV communication range. We define our MUAV as M={1,2,3,…,M} and LUAVs as a set of L={1,2,3,…,L}, where M≫L. LUAVs lie under the service of GBS, and MUAVs receive their bandwidth resources from LUAVs. MUAVs, deployed close to the primary observation scene, capture video content and transmit it to LUAVs, which then process the data and forward them to the edge server at GBS. The edge server performs the real-time processing of video, distributes it to the users, and allocates sufficient bandwidth to ensure smooth video streaming to the collaborative users. It is important to highlight that our objective is to minimize the transmission time between the observational scene and the collaborating users to ensure that each user receives similar information at the same time.

To enhance clarity, Table 1 summarizes the frequently used notations related to the system model.

For analysis, it is assumed that the UAV-based video streaming system follows the time slot structure, where the time *T* is discretized into *N* equal time slots. Although in a real-world scenario, video requests are typically generated following a probabilistic model reflecting user behavior, for simplicity, it is assumed that each user generates a video request at the beginning of time slot *t*, and any video request that is not received within slot *t* is considered video jamming. In the subsequent time slot, users generate new video requests. The duration of each time slot *t* is calculated as t=T/N. Each *t* being significantly small, the position of the MUAV and LUAV can be regarded as constant. However, the position of the users, LUAVs, and MUAVs may vary between time slots. Thus, at any time slot *t*, the service control decision of the GBS involves selecting LUAVs, allocating bandwidth, assigning video trans-rated ratios, and managing LUAV trajectories. Following the selection of the LUAV, MUAVs are associated with users based on the order in which they send data. This assignment follows a first-come, first-served (FCFS) policy, where the LUAV prioritizes MUAVs according to the sequence of data transmission. We assume that each user can only be served by one LUAV and one MUAV at a given time slot *t*. Therefore, we introduce a binary assignment variable $\beta_{t}^{u,l}$, similar to [20], to denote whether a video request from user *u* is assigned to connect with LUAV *l* for receiving data at time slot *t*. If the $u^{th}$ user is assigned to $l^{th}$ LUAV, $\beta_{t}^{u,l}$ is 1; otherwise, $\beta_{t}^{u,l}$ is 0. This binary variable plays a key role in modeling the assignment of users to specific UAVs for video streaming services, enabling efficient resource allocation within the system.

UAVs being freely moving objects, their positions are defined in the 3D coordinate system along with their respective velocities. Let the position of any $m^{th}$ MUAV be defined as $p_{t}^{m}=\left(x_{t}^{m},y_{t}^{m},h_{t}^{m}\right)\in R^{3X1}\forall m\in M$, having a velocity of $v_{t}^{m}$. For any $l^{th}$ LUAV, the position at any time slot *t* is defined as $p_{t}^{l}=\left(x_{t}^{l},y_{t}^{l},h_{t}^{l}\right)\in R^{3X1}\forall l\in L$ having velocity $v_{t}^{l}$. The velocities of MUAV and LUAV significantly impact the data transfer rate and transmission time from the the observational source to the end users. We can obtain the position of MUAV *m*, LUAV *l*, and user *u* at the next time slot as given in [4]:

(1) $$p_{t+1}^{m}=p_{t}^{m}+\left(1-\varrho \right)*v_{t}^{m}$$

(2) $$p_{t+1}^{l}=p_{t}^{l}+\left(1-\varrho \right)*v_{t}^{l}$$

(3) $$p_{t+1}^{u}=p_{t}^{u}+\left(1-\varrho \right)*v_{t}^{u}$$

where ϱ is the damping coefficient, which is added to prevent sudden changes in velocity for the MUAVs, LUAVs, and users. However, if any user is stationary, $v_{t}^{u}=0$. The distance of any $u^{th}$ user requesting service to $g^{th}$ GBS can be calculated using the Bray–Curtis distance [21] as

(4) $$d_{t}^{g,u}=\frac{|\left(x_{t}^{g}-x_{t}^{u}\right)+\left(y_{t}^{g}-y_{t}^{u}\right)|}{\left(x_{t}^{g}+x_{t}^{u}\right)+\left(y_{t}^{g}+y_{t}^{u}\right)},\forall u\in U,\forall g\in G$$

The distance between any $l^{th}$ LUAV and the $g^{th}$ GBS can be calculated [21] as

(5) $$d_{t}^{l,g}=\frac{|\left(x_{t}^{l}-x_{t}^{g}\right)+\left(y_{t}^{l}-y_{t}^{g}\right)|}{\left(x_{t}^{l}+x_{t}^{g}\right)+\left(y_{t}^{l}+y_{t}^{g}\right)},\forall l\in L\forall g\in G$$

And the distance between any $m^{th}$ MUAV and the $l^{th}$ LUAV can be calculated [21] as

(6) $$d_{t}^{m,l}=\frac{|\left(x_{t}^{m}-x_{t}^{l}\right)+\left(y_{t}^{m}-y_{t}^{l}\right)|}{\left(x_{t}^{m}+x_{t}^{l}\right)+\left(y_{t}^{m}+y_{t}^{l}\right)},\forall u\in U,\forall l\in L$$

### 2.1. Channel Condition Modeling

The wireless communication channels connecting users to the cooperative communication network play a pivotal role in shaping the efficiency and reliability of information exchange. Channel conditions, characterized by factors such as the Signal-to-Noise Ratio (SNR), path loss, fading, and interference, have a profound impact on the quality of communication and the overall performance of the network. As the communication between the GBS and users is subjected to terrain and obstacles, we opted for the Okumura–Hata path loss model [22], which is specifically designed for handling losses when a non-line-of-sight condition exists. Therefore, considering the channel gain being $\Phi_{1}$ from the Okumura–Hata path loss model [22], we calculate the channel fading $f_{t}^{g,u}$ between any user u∈U and $g^{th}$ GBS as

(7) $$f_{t}^{g,u}=\Phi_{1}*{\left(d_{t}^{g,u}\right)}^{-2},\forall u\in U,\forall g\in G$$

where $d_{t}^{g,u}$ is the distance between user *u* and the nearest GBS *g* from Equation (4).

In addition to the channel fading model as seen in Equation (4), the communication link from the LUAV to GBS is also subjected to atmospheric attenuation due to the absorption, scattering, and refraction of the signals. Therefore, an additional atmospheric attenuation model [23] is added with the channel fading $f_{t}^{l,g}$ between the $l^{th}$ LUAV and the $g^{th}$ GBS, which is calculated as

(8) $$f_{t}^{l,g}=\Phi_{2}*{\left(d_{t}^{l,g}\right)}^{-2}*e^{\alpha_{1}*d_{t}^{l,g}},\forall l\in L\forall g\in G$$

where $d_{t}^{l,g}$ is the distance between LUAV *l* and nearest GBS *g* from Equation (5), $\Phi_{2}$ is the channel gain from the Okumura–Hata path loss model, and $\alpha_{1}$ is the atmospheric attenuation coefficient.

Moreover, as demonstrated in Figure 2, any $m^{th}$ MUAV can offload the original $k^{th}$ video directly to one LUAV *l* at a time. As UAVs operate in free space, we assume minimal interference exists during video offloading between the MUAV-LUAV and the communication link characterized by a direct line of sight. Therefore, the channel fading between the $m^{th}$ MUAV and the $l^{th}$ LUAV, $f_{t}^{m,l}$, can be defined using the free-space path loss model, which can be calculated as in [7]

(9) $$f_{t}^{m,l}=\Phi_{3}*{\left(d_{t}^{m,l}\right)}^{-2}*e^{\alpha_{2}*d_{t}^{m,l}},\forall m\in M,\forall l\in L$$

where $\Phi_{2}$ represents the channel power gain per unit distance, and $\alpha_{2}$ is the atmospheric attenuation coefficient between MUAVs and LUAVs.

### 2.2. Bandwidth Condition Modeling

In the real world, the user in collaboration requests the nearest edge server for real-time video streaming. The request mainly involves a video stream identifier to indicate which live stream the user wants, the user authentication information, and network constraints, including information related to the network congestion, currently available bandwidth, or the probable data rate. Therefore, the size of the information in these requests is small. As a result, the bandwidth required to transmit these requests is minimal. However, the live stream video size is large, even after video transcoding. Therefore, in our case, the edge server needs to distribute the available bandwidth only for downloading the video at the user’s end and uploading the video at the LUAV and MUAV. Let the total bandwidth available for the $g^{th}$ GBS during any time slot *t* be denoted as $B_{t}^{g}$.

If any g∈G allocates $B_{t}^{u}$ and $B_{t}^{l}$ among the users and link UAVs, the total allocated bandwidth of the users and LUAV should satisfy the following:

(10) $$B_{t}^{g}=\sum_{u\in U}B_{t}^{u}+\sum_{l\in L}B_{t}^{l}$$

We further assume that all the MUAVs, LUAVs, and users operate in an orthogonal frequency division multiple access (OFDMA) mode. Since only downlink transmission occurs between the GBS and users, the video transfer rate from the $g^{th}$ GBS to $u^{th}$ collaborative user for any $k^{th}$ video segment at time slot *t* can be calculated [4] as

(11) $$\Psi_{t}^{g,u}=B_{t}^{u}*\log_{2}\left(1+\frac{\rho_{t}^{g,u}f_{t}^{g,u}}{\chi_{1}}\right)$$

where $\rho_{t}^{g,u}$ is the transmit power between user *u* and GBS *g*; $f_{t}^{g,u}$ is the value of channel fading model obtained from (7); and $\chi_{1}$ is the additive white Gaussian noise at the user. However, it is to be noted that the bandwidth allocation for both the UAVs, LUAV and MUAV, requires uplink transmission, and the MUAV will transfer the data to LUAV, and the LUAV will upload the received data to GBS. The video transmission rate from the LUAV to GBS for any $k^{th}$ video segment at time slot *t* is calculated [4] as

(12) $$\Psi_{t}^{l,g}=B_{t}^{l}*\log_{2}\left(1+\frac{\rho_{t}^{l,g}f_{t}^{l,g}}{\chi_{2}}\right)$$

while the available bandwidth at $l^{th}\in L$ is divided equally among *M* MUAVs to ensure fairness among them. As equal sharing guarantees, each MUAV receives an equal share of available resources to transmit their observed data. Moreover, equal sharing facilitates load balancing among the MUAVs and prevents the MUAVs from being unfairly disadvantaged in terms of bandwidth access. Therefore, the video transfer rate for the uplink transmission of any *k* video segment between MUAV and LUAV can be calculated [2,4] as

(13) $$\Psi_{t}^{m,l}=\frac{B_{t}^{l}}{M^{\prime }+1}*\log_{2}\left(1+\frac{\rho_{t}^{m,l}f_{t}^{m,l}}{\varpi_{t}^{m,l}+\chi_{3}}\right)$$

where $\varpi_{t}^{m,l}$ represents the interference experienced by the $m^{th}$ MUAV receiving services from the $l^{th}$ LUAV during any time stamp *t*. $M^{\prime }\subset M$, which depends on the number of MUAVs receiving bandwidth services from $l^{th}$ LUAV. $\chi_{2}$ and $\chi_{3}$ are the additive white Gaussian noise. And $\rho_{t}^{u,g}$ and $\rho_{t}^{m,l}$ are the transmit power of the LUAV and MUAV, respectively.

### 2.3. Delay Modeling

The delay during video streaming can have negative impacts on synchronization, engagement, and decision-making among the collaborating users. To ensure a high QoE for all users, it is important to minimize transmission delays. This study identifies the different stages of transmission delay, including the video transmission delay from the MUAV to LUAV, video transmission delay at the LUAV, video transmission delay from the LUAV to GBS, video processing delay at the GBS, and the video transmission delay from the GBS to the user. Therefore, for any $u^{th}$ user, the total delay can be calculated as

(14) $$T_{t}^{u,g}=T_{\zeta ,t}^{u,g}+T_{\zeta ,t}^{g,l}+T_{\zeta ,t}^{l,m}+T_{t}^{m,l}+T_{t}^{l,g}+T_{t}^{g,u}$$

where $T_{\zeta ,t}^{u,g}$, $T_{\zeta ,t}^{g,l}$, and $T_{\zeta ,t}^{l,m}$ are negligible because the size of the video request data from the user to the GBS or from the GBS to UAVs is very small. Thus, the request transmission delay from the user to the GBS and the GBS to UAVS can be ignored. We, therefore, introduce the $T_{t}^{m,l}$, $T_{t}^{l,g},T_{t}^{g,u}$ to denote the transmission delay from MUAV to LUAV, LUAV to GBS, and GBS to users in the context of our study. MUAV *m* directly transfers the original video without any processing to the $l^{th}$ LUAV during *t*. Therefore, the transmission time $T_{t}^{m,l}$ can be defined as

(15) $$T_{t}^{m,l}=\frac{D_{t}^{k}}{\Psi_{t}^{m,l}q_{t}^{m}}$$

where $D_{t}^{k}$ is the original video data size for the $k^{th}$ video sequence, and $q_{t}^{m}$ is the CPU-related parameters of the MUAV *m* at time slot *t*.

The LUAV on receiving the data from MUAV performs video trans-rating, whose main purpose is to adjust the resolution of the video based on the available bandwidth such that the time for the transmission is minimized without much degradation of the video quality. Thus, the video trans-rating process is an essential yet computationally intensive task that involves the processing delay and subsequently the queuing delay in addition to the transmission delay from the LUAV to the GBS. The processing delay is inherent to the data size and computational capacity of the CPU at the LUAV. Assuming the size of the $k^{th}$ video data before trans-rating is $D_{t}^{k}$, we introduced a set of the time-varying video trans-rated ratio $\Phi_{t}^{l,g}\in \left[0,1\right]$. $\Phi_{t}^{l,g}$ will help to reduce the size of the received video during transmission and in turn help in reducing the transmission delay. The processing delay is calculated as

(16) $$T_{t,C}^{l}=\frac{D_{t}^{k}\Phi_{t}^{l,g}}{q_{t}^{l}}$$

where $D^{k}\Phi_{t}^{l,g}$ is the trans-rated video data size, and $q_{t}^{l}$ is the CPU-related parameters of the LUAV *l* during the current time cycle. As a result of the processing delay, this trans-rating process incurs some queuing delay, which is characterized by the arrival rate of the video frames, the computation rate of the LUAV, and the length of the queue in the LUAV. Considering the video arrival rate at $l^{th}$ LUAV from Equation (13) as $\Psi_{t}^{m,l}$, the queuing delay at the $l^{th}$ LUAV can be calculated with the help of Little’s Law [24] as

(17) $$T_{t,Q}^{l}=\frac{\Psi_{t}^{m,l}}{q_{t}^{l}\left(q_{t}^{l}-\Psi_{t}^{m,l}\right)}$$

where $q_{t}^{l}$ is the CPU-related parameters of the LUAV *l* at time slot *t*.

The transferring delay $T_{t,E}^{l}$ due to the transmission rate of $\Psi_{t}^{l,g}$ can be calculated as

(18) $$T_{t,E}^{l}=\frac{D_{t}^{k}\Phi_{t}^{l,g}}{\Psi_{t}^{l,g}},\forall l\in L$$

Thus, the total transmission time from LUAV *l* to GSB *g* can be calculated as

(19) $$T_{t}^{l,g}=T_{t,C}^{l}+T_{t,Q}^{l}+T_{t,E}^{l}=\frac{D_{t}^{k}\Phi_{t}^{l,g}}{q_{t}^{l}}+\frac{\Psi_{t}^{m,l}}{q_{t}^{l}\left(q_{t}^{l}-\Psi_{t}^{m,l}\right)}+\frac{D_{t}^{\prime k}\Phi_{t}^{l,g}}{\Psi_{t}^{l,g}}$$

However, in the case of severe scarcity in the bandwidth, a GBS can choose to further trans-rate the received data from LUAV *l*. Let the trans-rate ratio at GBS be defined as $\Phi_{t}^{g,u}\in \left[0,1\right]$, which will reduce the processing delay by a factor of $\Phi_{t}^{g,u}$. Therefore, the transmission delay for any $u^{th}$ user from $g^{th}$ GBS can be calculated as

(20) $$T_{t}^{g,u}=T_{t,C}^{g,u}+T_{t,Q}^{g,u}+T_{t,E}^{g,u}=\frac{D_{t}^{\prime k}\Phi_{t}^{g,u}}{q_{t}^{g}}+\frac{\Psi_{t}^{g,u}}{q_{t}^{g}\left(q_{t}^{g}-\Psi_{t}^{g,u}\right)}+\frac{D_{t}^{\prime k}\Phi_{t}^{g,u}}{\Psi_{t}^{u,g}}$$

where $T_{t,C}^{g,u}$, $T_{t,Q}^{g,u}$, and $T_{t,E}^{g,u}$ are the processing delay, queuing delay and transferring delay at the $g^{th}$ GBS for the $u^{th}$ user at any time slot *t* and $D_{t}^{\prime k}=D_{t}^{k}\Phi_{t}^{l,g}$.

### 2.4. Fairness Modeling

In our scenario, users collaborate during emergencies, and they may simultaneously request the same video data. Each user requires a specific bandwidth allocation to receive critical time-varying video streams. Similarly, each LUAV requires bandwidth allocation in order to provide network resources to the MUAVs and upload video data streams from the LUAV to the GBS. If the available bandwidth $B_{t}^{u}$ at the GBS is shared equally among users, the transmission time $T_{t}^{g,u}$ for certain users u∈U may potentially exceed permissible deadlines δ due to the varied user locations and channel conditions. This can lead to issues like video stuttering, loss of video frames, or delayed response for the users.

To ensure equitable resource distribution and a satisfactory user experience, our goal is to minimize the standard deviation of total delay for each user u∈U. This approach aims to achieve fairness in QoE by ensuring that all users receive information within consistent time frames during each time slot. Quantifying fairness among |U| users involves evaluating the standard deviation of total delay during video streaming. The fairness among the users, $F_{1}\left(U\right)$, is expressed as

(21) $$F_{1}\left(U\right)=\sqrt{\frac{1}{\left|U\right|}\sum_{u=1}^{U}{\left(T_{t}^{u,g}-\frac{1}{\left|U\right|}\sum_{u=1}^{U}T_{t}^{u,g}\right)}^{2}}$$

where $T_{t}^{u,g}$ is the total delay obtained from Equation (14). Similarly, the fairness among the LUAVs can be expressed as

(22) $$F_{2}\left(L\right)=\sqrt{\frac{1}{\left|L\right|}\sum_{l=1}^{L}{\left(T_{t}^{l,g}-\frac{1}{\left|L\right|}\sum_{l=1}^{L}T_{t}^{l,g}\right)}^{2}}$$

where $T_{t}^{l,g}$ is the total transmission time from the $l^{th}$ LUAV to the $g^{th}$ GBS obtained from Equation (19).

### 2.5. Priority Modeling

In real emergency scenarios, multiple dynamic factors affect the overall latency and QoE of the users. Those dynamic factors can be attributed to varying channel conditions, the movement of the users, and current available resources at the edge servers. As the position of the users will vary based on the emergency, we want to emphasize the fairness of edge resource access to all collaborating users including LUAVs. Therefore, we assigned priority to collaborating users exponentially as

(23) $$\xi_{t}^{u,g}=e^{-\kappa_{u}d_{t}^{u,g}}\forall u\in U$$

However, in the case of LUAVs, the priority is measured in terms of the average accumulated user assignment for the LUAV at the GBS as shown in

(24) $$req_{t}^{l}=\frac{1}{\left|U\right|}\sum_{i=1}^{\left|U\right|}\beta_{t}^{i,l}$$

(25) $$\xi_{t}^{l,g}=e^{-\kappa_{l}req_{t}^{l}}\forall l\in L$$

where $\kappa_{u}$ and $\kappa_{l}$ are positive weighted decay constants, and $d_{t}^{u,g}$ indicates the spatial distance between the $u^{th}$ user and the $g^{th}$ GBS, and $d_{t}^{l,g}$ indicates the spatial distance between the $u^{th}$ user and the $g^{th}$ GBS.

## 3. Problem Formulation

Section 2 provids an overview of the scenario under consideration, including the impact of channel conditions on bandwidth distribution and total transmission delays across MUAVs, LUAVs, GBS, and users. This paper is dedicated to designing an adaptive resource management strategy for UAV-based video data streaming during emergencies. Our goal is to minimize the total delay time through adaptive bandwidth allocation among users, LUAVs, and MUAVs, incorporating video compression and trajectory control of LUAVs. This section begins by introducing the relevant parameters and factors involved in the system. Subsequently, it derives the final QoE function that encapsulates the performance objectives and optimization criteria for the proposed adaptive resource management strategy.

### 3.1. Minimization of the Average Total Delay for Users

Minimization of the average total delay experienced by |U| users can be achieved through the strategic selection of LUAVs positioned close to users. This approach not only reduces individual user delay but also contributes towards minimizing the standard deviation among collaborating users. The average total delay for users can be defined as

(26) $$min\left(\frac{1}{\left|U\right|}\sum_{u=1}^{U}T_{t}^{u,g}\right)$$

such that the following hold:

(i) $\sum_{u=1}^{U}B_{t}^{u}+\sum_{l=1}^{L}B_{t}^{l}\leq B_{t}^{g}$, ∀u∈U, ∀l∈L, ∀g∈G.
(ii) $\Phi_{t}^{g,u}\leq \Phi_{t,max}^{g,u}$, ∀u∈U, ∀g∈G, where $\Phi_{t,max}^{g,u}$ is the maximum allowable video trans-ration.
(iii) $\Phi_{t}^{l,g}\leq \Phi_{t,max}^{l,g}$, ∀l∈L, ∀g∈G, where $\Phi_{t,max}^{l,g}$ is the maximum allowable video trans-ration.
(iv) $q_{t}^{l},q_{t}^{m}>0$ to ensure that both LUAVs and the GBS have available computational resources.
(v) $T_{t}^{u,g}$ ≤ δ ∀u∈U.
(vi) $d_{t}^{m,l}$$\geq d_{min}$ where $d_{min}$ is the safest allowable distance to avoid collision between the MUAV and the LUAV.

### 3.2. Average Transmission Time Minimization of LUAVs

Minimization of the average transmission time experienced by |L| LUAVs can be achieved with a better transmission rate which can be achieved through better bandwidth assignment and video trans-ration. By achieving better bandwidth assignment and optimizing video transcoding, we can reduce the transmission time for LUAVs, consequently reducing the average delay experienced by users. The average total transmission time for LUAVs can be mathematically described as follows:

(27) $$min\left(\frac{1}{\left|L\right|}\sum_{l=1}^{L}T_{t}^{l,g}\right)$$

such that conditions (*i*), (*iii*), (*iv*), (*vi*) hold true.

### 3.3. Maximization of Fairness among Users and LUAVs

To maintain stable and satisfactory QoE for users receiving video streams from the nearest GBS, it is crucial to maximize fairness among collaborating users and LUAVs. Fairness among users can be achieved by minimizing disparities in the total delay experienced during collaboration. Similarly, fairness among LUAVs involves minimizing the total transmission time between LUAVs and the GBS. Integrating these fairness objectives for both users and LUAVs yields the following representation:

(28) $$min\left(F_{1}\left(U\right)\right)+min\left(F_{2}\left(L\right)\right)$$

such that conditions (i),(ii),(iii),(iv),(v),(vi) hold true.

### 3.4. Minimization of Video Trans-Rates among Users and LUAVs

In UAV-based video streaming systems, ensuring high video quality is essential for delivering a satisfactory user experience. However, achieving superior video quality often entails managing large data sizes, necessitating substantial bandwidth resources. Therefore, optimizing video quality involves adjusting the video trans-rated ratio both at LUAV and GBS. We used the relative difference in the video trans-rated ratio to quantify that the video compression with 1 signifies no video trans-ration. The average relative video quality estimation is described in

(29) $$\min(P\left(\Phi_{t}^{l,g},\Phi_{t}^{g,u}\right))$$

where the value of $P(\Phi_{t}^{l,g},\Phi_{t}^{g,u})$ is calculated as

(30) $$P\left(\Phi_{t}^{l,g},\Phi_{t}^{g,u}\right)=\frac{1}{\left|L\right|}\sum_{l=1}^{\left|L\right|}\left(1-\Phi_{t}^{l,g}\right)+\frac{1}{\left|U\right|}\sum_{u=1}^{\left|U\right|}\left(1-\Phi_{t}^{g,u}\right)$$

such that conditions (i),(ii),(iii),(iv),(v),(vi) hold true.

### 3.5. QoE of the UAV-Based Video Streaming System

We formulate the QoE of the system at any time slot based on the minimization of the average total delay for users together with the fairness among users and LUAVs along with the minimization of the video trans-ration for both users and LUAVs. The QoE function $\Gamma_{t}$ for any time slot *t* is defined as

(31) $$\Gamma_{t}=\lambda_{1}\cdot \left\{\left(\frac{1}{\left|U\right|}\sum_{u=1}^{U}T_{t}^{u,g}\right)+\left(\frac{1}{\left|L\right|}\sum_{l=1}^{L}T_{t}^{l,g}\right)+F_{1}\left(U\right)+F_{2}\left(L\right)\right\}+\lambda_{2}\cdot P\left(\Phi_{t}^{l,g},\Phi_{t}^{g,u}\right)$$

where $\lambda_{1}+\lambda_{2}=1$ and $\lambda_{1}$,$\lambda_{2}$ are dimensionless weighting factors.

We describe our objective function as in Equation (32), where $\Gamma_{t}$ represents the overall QoE of the UAV-based video streaming system during emergencies:

(32) $$\mathrm{Minimize}:\;\sum_{t=1}^{N}\Gamma_{t}$$

such that (i),(ii),(iii),(iv),(v),(vi) hold true.

## 4. Deep Reinforcement Learning-Based Approach

The dynamic optimization problem described in Equation (32) involves making sequential decisions to optimize the objective under given constraints. Traditional resource allocation methods, such as static optimization and game theory, face difficulties in handling this type of problem. These approaches typically focus on finding near-optimal policies by maximizing immediate rewards based on the current state. In the following sections, we reformulate the problem described by Equation (32) as a Markov Decision Process (MDP) and then apply the Deep Deterministic Policy Gradient (DDPG) algorithm to address the MDP.

### 4.1. MDP Components

In general, the MDP is described by the tuple 〈S,A,F,r,γ〉, where S represents the set of observable states in the environment, A denotes the possible actions available to agents, F indicates the transition function defined as F:SXA→S, and r indicates the rewards received by agents upon taking a specific action, r:SXA→R following any policy π. Additionally, γ stands for the discount factor, which determines the influence of future rewards. The policy π can be described as π:S→R to indicate mapping from *s* to *a*. In summary, the RL agent observes the environment states s∈S and performs action a∈A following policy π and receives reward *r*. The environment transitions to a new state $s^{\prime }$, and the agent receives a new reward. This cycle repeats until the current episode concludes.

In our case, the agent represents any GBS g∈G whose primary objective is to allocate bandwidth among UAVs and users, while optimizing the video transfer rate to uphold satisfactory video quality by maximizing the expected cumulative reward. The characteristics of our environment, including its states, actions, and rewards, are described as follows:

1. State space: The positions of the MUAV, LUAV, and users are changing constantly. Thus, GBS needs to constantly adjust the available bandwidth, available computational resources, and video trans-ration factor along with the velocities of MUAV and LUAV. We analyze the state space from the following aspects:
  (a) Priority: The priority of each user and LUAVs is distance dependent and can be obtained from Equations (23) and (25).
  (b) Location information: The current location of the MUAV, LUAV, and users obtained from Equations (1)–(3).
  (c) Channel state information: The video transfer rate between the GBS to the users, the LUAV to the GBS, and the MUAV to the LUAV, obtained from Equations (11), (12), and (13), respectively.
  (d) Bandwidth allocation: The currently allocated bandwidth, $B_{t}^{u}$ and $B_{t}^{l}$, ∀u∈U,∀l∈L between users and LUAV as the available bandwidth at any GBS is shared for decision-making.
  (e) Video trans-ration ratio: The current video trans-ration ratio between the LUAV to the GBS, and the GBS to users $\Phi_{t}^{l,g}$,$\Phi_{t}^{g,u}$.
2. Action space: The action space describes the following information pertinent to action $a_{t}$:
  (a) Selection of LUAVs: The nearest LUAV is selected based on the priority obtained from Equation (21).
  (b) Velocity adjustment: The adjustment of the velocities of LUAVs helps in maintaining the standard video transmission rate and also helps in minimizing the transmission time. The velocity at the next time slot can be calculated using Equation (3).
  (c) Bandwidth adjustment: The bandwidth is initially adjusted through time-varying modulation components $\Omega_{t}^{u}$ and $\Omega_{t}^{l}$ such that
    (33) $$\sum_{i=1}^{U}\left(\Omega_{t}^{i}\cdot Bt^{i}\right)+\sum_{j=1}^{\left|L\right|}\left(\Omega_{t}^{j}\cdot B_{t}^{j}\right)=B_{t}^{g}$$
    where $\Omega_{t}^{u}$ and $\Omega_{t}^{l}$ are modulation components in the range (0,1). Here, *i* represents each user in the set of users U, that is, $\left\{\Omega_{t}^{1},\Omega_{t}^{2},\ldots \Omega_{t}^{\left|U\right|}\right\}$. However, it is to be noted that the DDPG generates an action value varying between 0 and 1 for the bandwidth allocation constrained by Softmax. If $\Omega_{t}^{i}=0$ or $\Omega_{t}^{j}=0$, then the previous value is retained.
  (d) Video trans-ration ratio adjustment: The value of $\Phi_{t}^{l,g}=1$ or $\Phi_{t}^{g,u}=1$ means no trans-ration while $\Phi_{t}^{l,i}=\frac{1}{16}$ indicates maximum downsampling of the video, that is, 6.25% of the original size. The value of the video trans-ration ratio for both LUAVs and users are treated as time-varying components that satisfy the inequality $0<\Phi_{t}^{l,g},\Phi_{t}^{g,u}\leq 1$
3. Reward: The agent g∈G will be rewarded for reducing the standard deviation of the transmission time as well as for avoiding excessive video trans-ration penalties described in Equation (32). The goal of *g* is to maximize the value of $r_{t}^{a}$ for any state $s_{t}$ and action $a_{t}$, which can be defined as follows:
  (34) $$r_{t}^{a}=-\left(\sum_{t=1}^{N}\Gamma_{t}+r_{t,c}\right)$$
  Here, $r_{t,c}$ signifies the in-bound penalty which occurs when the distance between the MUAV and the LUAV falls bellow the minimum allowable distance $d_{min}$, that is, $d_{t}^{m,l}\leq$ $d_{min}$. Therefore, we define $r_{t,c}$ = 0 if $d_{t}^{m,l}\geq$ $d_{min}$; Otherwise, $r_{t,c}$ incurs a negative penalty of −5.

### 4.2. DDPG-Aided Video Data Streaming Algorithm

In the proposed MECS environment, the edge server at the GBS distributes its communication resources among the LUAVs and users working cooperatively. The video arrival rate at the GBS is unpredictable, and the wireless communication medium is time varying. In such scenarios, DRL-based algorithms become desirable, as they try to provide the optimal bandwidth allocation by considering a series of unavoidable constraints, such as the distance of the users from the GBS, the location and moving speed of the users, and the wireless channel conditions along with the relaying capacities of the LUAVs. Additionally, the LUAVs are also required to maintain optimal velocities while relaying to mitigate longer video transmission times. DRL methods can be designed for both discrete and continuous state–action spaces. As discussed in Section 2.1, our state–action space is continuous in nature, and thus the DDPG-based algorithm became our first choice. We implemented the DDPG-based Video Data Streaming Algorithm (DVDA) to deliver responses during emergencies. This ensures that all users (emergency responders) receive the same video information simultaneously while maintaining a balanced distribution of networking resources to the LUAVs. The DVDA is a policy gradient algorithm that computes the expected value without the need for optimal action selection, making it suitable for continuous state–action spaces. Moreover, the DVDA operates off-policy, allowing UAVs to train using offline data, which is advantageous. Additionally, the DVDA follows a deterministic policy, meaning that the same policy consistently selects the same action in a given state. Consequently, the DVDA algorithm offers enhanced learning efficiency, improved convergence, and greater stability. The algorithm for the join bandwidth allocation, video trans-ration, and trajectory control is defined in Algorithm 1.

The DVDA framework incorporates two essential components: the policy function and the Q-value function. The policy function, serving as the actor, determines the actions to be taken in a given state, while the Q-value function, serving as the critic, evaluates the quality of those actions. In other words, the actor generates actions, while the critic evaluates the actor’s performance and guides its subsequent actions. In simpler terms, the DVDA consists of two deep neural networks: an actor network and a critic network. The actor-network $\mu (s|\theta^{\mu })$ represents the policy function, and the critic network $Q(s,a|\theta^{Q})$ represents the Q-value function, where $\theta^{\mu }$ and $\theta^{Q}$ indicates the parameters of the actor and critic networks respectively. To stabilize training and improve convergence, both the actor and critic networks include corresponding target networks, denoted as $\mu^{\prime }$ with network parameters of $\theta^{\mu }$ and $Q^{\prime }$ with network parameters $\theta^{Q^{\prime }}$ respectively. On the other hand, the critic network $Q(s,a|\theta^{Q})$ is trained to approximate the Q-value function, which estimates the expected return by taking action *a* in state *s*, following action μ.
**Algorithm 1** DVDA algorithm for bandwidth allocation, video trans-rates along with trajectory control  1:Set the initial network weights $\theta^{\mu }$, $\theta^{Q}$, $\theta^{\mu^{\prime }}$, $\theta^{Q^{\prime }}$  2:Clear the experience replay buffer $N_{B}$  3:Initialize the discount factor γ  4:Initialize the Gaussian distribution-based noise parameters: $\mu_{e}$ and $\sigma_{e}$  5:Initialize the maximum number of episodes: *E*  6:**for** each episode e=1∈E **do**  7:   Reset the system’s simulation parameters and obtain the initial observation state, $s_{1}$  8:   **for** t = 1 to T **do**  9:       Normalize state $s_{t}$ to $\hat{s_{t}}$10:      Retrieve the action using $\theta^{\mu }$ and execute action $a_{t}$ with noise $\varphi_{t}\approx N\left(\mu_{e,t},\sigma_{e,t}\right)$11:      Obtain the reward $r_{t}^{a}$ according to Equation (34)12:      Observe the next state $s_{t+1}$ and normalize it to $\hat{s_{t+1}}$13:      **if** $N_{B}$ not full **then**14:         Save the transition $\left(\hat{s_{t}},a_{t},r_{t}^{a},\hat{s_{t+1}}\right)$ to replay buffer $N_{B}$15:      **else**16:         Remove any 1 transition in replay buffer $N_{B}$ with $\left(\hat{s_{t}},a_{t},r_{t}^{a},\hat{s_{t+1}}\right)$17:         Sample any $N_{I}$ transitions,$\left(\hat{s_{i}},a_{i},r_{i}^{a},\hat{s_{i+1}}\right)\forall i=1,2,\ldots ,N_{I}$ from $N_{B}$ for training actor and critic network18:         Compute $\theta^{Q^{\prime }}$ ’s gradient according to Equation (36)19:         Update the weights $\theta^{Q}$ of critic network using Adam optimizer20:         Obtain $\theta^{Q}$’s policy gradient using Equation (31)21:         Update the weights of $\theta^{\mu }$ with Adam optimizer22:         Use $\tau_{0}=0.02$ to update $\theta^{\mu^{\prime }}$ and $\theta^{Q^{\prime }}$ utilizing Equations (37) and (38)23:      **end if**24:   **end for**25:**end for**

Following [25], the actor-network can be trained to maximize the expected return by updating its parameters, $\theta^{\mu }$ using the policy gradient:

(35) $$\nabla_{\theta^{\mu }}J\approx E_{s}\left[\nabla_{\theta^{\mu }}\mu \left(s|\theta^{\mu }\right)\nabla_{a}Q\left(s,a|\theta^{Q}\right){|}_{s=s_{t},a=\mu (s_{t}|\theta^{\mu })}\right]$$

Similarly, the critic network can be trained to minimize the Mean Squared Error (MSE) loss between the predicted Q-values and the target Q-values obtained from the Bellman equation [25]:

(36) $$L\left(\theta^{Q}\right)=E\left[{\left(r_{t}^{a}+\gamma Q\left(s^{\prime },\mu^{\prime }\left(s^{\prime }|\theta^{\mu^{\prime }}\right)|\theta^{Q^{\prime }}\right){|}_{s^{\prime }=s_{t^{\prime }+1}}-Q\left(s,a|\theta^{Q}\right){|}_{a=a_{t},s=s_{t}}\right)}^{2}\right]$$

where $r_{t}^{a}$ is the reward received after taking action *a* in state *s*, $s^{\prime }$ is the next state, γ is the discount factor, and $\mu^{\prime }$ and $Q^{\prime }$ represent the target actor and critic networks, respectively.

The training procedure of the DVDA algorithm encompasses the following steps. Initially, the actor network, μ, generates output $\mu (s_{t})$ following the preceding training iteration. To ensure comprehensive exploration of the state space, it’s imperative to strike a balance between exploration and exploitation. Notably, exploration in DVDA can be treated independently of the learning process since DVDA operates as an off-policy algorithm. Consequently, we construct the action space by introducing behavior noise $\varphi_{t}$, resulting in actions $a_{t}=\mu \left(s_{t}\right)+\varphi_{t}$, where $\varphi_{t}$ follows a Gaussian distribution $(\varphi_{t}\approx N\left(\mu_{e},\sigma_{e,t}\right)$ with mean $\mu_{e}$ and standard deviation $\sigma_{e,t}$. Upon execution in the environment, the agent observes the subsequent state $s_{t+1}$ and receives the immediate reward $r_{t}$. This transition, represented as $\left(s_{t},a_{t},r_{t},s_{t+1}\right)$ is stored in the experience replay buffer. Subsequently, *N* transitions $\left(s_{t^{\prime }},a_{t^{\prime }},r_{t^{\prime }},s_{t^{\prime }+1}\right)$ are randomly selected from the buffer to form a mini-batch, which is then fed into both the actor network and the critic network. Using this mini-batch, the actor target network $\mu_{0}$ outputs actions $\mu (s_{t}^{\prime })$ to the critic target network $Q_{\theta }$. With the mini-batch and $\mu (s_{t^{\prime }})$, the critic network calculates the target value, $y_{t}$ based on Equation (38).

The critic network *Q* can be modified with Adam optimizer for minimizing the loss function. Consequently, the actor network μ provides the minibatch action $a=\mu (s_{t})$ to the critic network to acquire the action’s gradient. Finally, the DVDA agent updates the actor target network and the critic target network using a small fixed value, $\tau_{0}$ as

(37) $$\theta^{\mu^{\prime }}\leftarrow \theta^{\mu }+\left(1-\tau_{0}\right)\theta^{\mu^{\prime }}$$

(38) $$\theta^{Q^{\prime }}\leftarrow \theta^{Q}+\left(1-\tau_{0}\right)\theta^{Q^{\prime }}$$

## 5. Performance Evaluation

In this section, the performance of the proposed algorithm for bandwidth allocation and video transmission with UAV trajectory control is evaluated. The initial step involves configuring simulation parameters. Subsequently, the efficacy of the DVDA framework is assessed across various scenarios and against alternative baseline schemes.

### 5.1. Simulation Setup

This study examines the effectiveness of UAV-assisted collaboration between edge servers and users in emergency situations to enhance collaborative performance. To investigate this, a basic system model is utilized to analyze the innovative approach. For initial model performance analysis a MUAV, a LUAV, a base station, and 5 users were chosen. The flight velocities for both MUAV and LUAV are uniformly distributed between 30 m/s and 60 m/s. The users’ speeds vary between 10 m/s and 20 m/s. However, the proposed scheme and algorithm can be extended to handle more complex scenarios involving multiple MUAVs, LUAVs, and users. Therefore, to highlight the effect of multiple users, 2 MUAVs were included under each LUAV. The number of LUAVs were set to 1, 2, 3, 4, 5 and number of users were set to 5, 15, 25, 35 and 45. GBS was located at [0,0,0] m. The initial location of the users was randomly generated between 10 m and 50 m. The bandwidth of the GBS varies between 100 KHz and 200 KHz. For both MUAV and LUAV, the noise power is uniformly distributed between −130 dBm and −140 dBm. For users, the noise power is uniformly distributed between −100 dBm and −120 dBm. The channel power gain per unit distance is kept at −50 dB. The size of video data at MUAV follows a uniform distribution between 70 Mbps and 100 Mbps. The CPU capacity of the GBS and edge server varies between 80 GHz and 100 GHz and 0.6 GHz to 2 GHz, respectively.

### 5.2. DVDA Model Configuration

The proposed DVDA contains an actor network and a critic network. he actor network comprises three fully connected hidden layers, each containing 400,300,100 neurons respectively with ReLU6 activation function that clips the output value at 6. The final output layer of the actor-network directly coincides with the action dimension where the action dimension is calculated as 3∗len(L)+3∗len(M), with softmax activation applied as the action value generator. The critic network, on the hand, consists of two fully connected dense layers with 300 and 100 neurons respectively. The final regression layer is a dense layer which outputs the *Q* value $Q(s_{t},a_{t})$. The number of epochs, *E* is set to 400 with number of episodes *T* is set to 200 in the training stage. Given the environment’s perpetual nature and continuous action policy, we opt to terminate the training process manually after each episode, subsequently resetting the environment. The training commences only once the buffer attains its full capacity, which is 5000 in our case. During the initial 5000 steps, a random policy governs the actions taken, with the training regime initiating at each subsequent step. The entire actor network and critic network have been developed using the Pytorch framework, with the learning rate for the actor network and critic network set to 0.0001 and 0.0002, respectively. The γ value is varied between 0 and 1.

### 5.3. Empirical Results and Interpretations

This section presents several empirical analyses, including the average reward system across different models, the impact of increasing user numbers on average user delay, the relationship between the number of LUAVs and the average LUAV transmission time, the assessment of fairness for both users and LUAVs, and the system’s average response under varying video data sizes and bandwidth allocations. The performance of the proposed DVDA algorithm is evaluated against the baseline DDPG without noise (DPDG-NN), the DDPG with the ‘tanh’ activation function (DDPG-T), the DDPG with the ‘softplus’ activation function (DDPG-S), and a baseline Actor–Critic (AC) method, using identical hyperparameters across all models.

#### 5.3.1. Impact of Network Parameters

**Figure 3:**
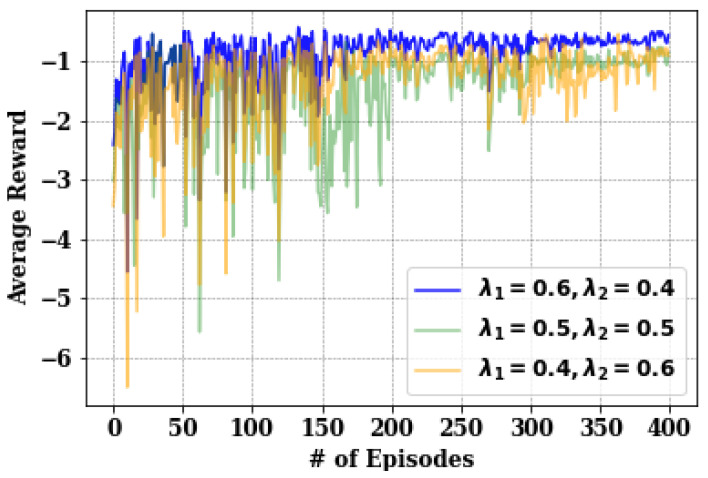
Comparison of different algorithms on the average reward of the system.

In the experiment, the network parameters correspond to the variation of the $\lambda_{1}$ and $\lambda_{2}$ values in the reward (Equation (34)) of DVDA. These parameters play a crucial role in determining the behavior of the reinforcement learning agent, the GBS. Specifically, $\lambda_{1}$ represents the relative importance of reducing the average transmission time for both the LUAVs and users along with minimizing the standard deviation of the transmission time for both the LUAVs and users, while $\lambda_{2}$ represents the relative importance of avoiding video trans-ration penalties. Different combinations of $\lambda_{1}$ and $\lambda_{2}$ were explored, including (0.6,0.4), (0.5,0.5), and (0.4,0.6). As shown in Figure 3, the combination of $\lambda_{1}=0.6$ and $\lambda_{2}=0.4$ yielded the best cumulative reward. This indicates that the agent achieved the highest cumulative reward when placing relatively more emphasis on reducing the standard deviation of the transmission time ($\lambda_{1}$) compared to avoiding video trans-ration penalties ($\lambda_{2}$).

#### 5.3.2. Impact on Average Delay of Users

**Figure 4:**
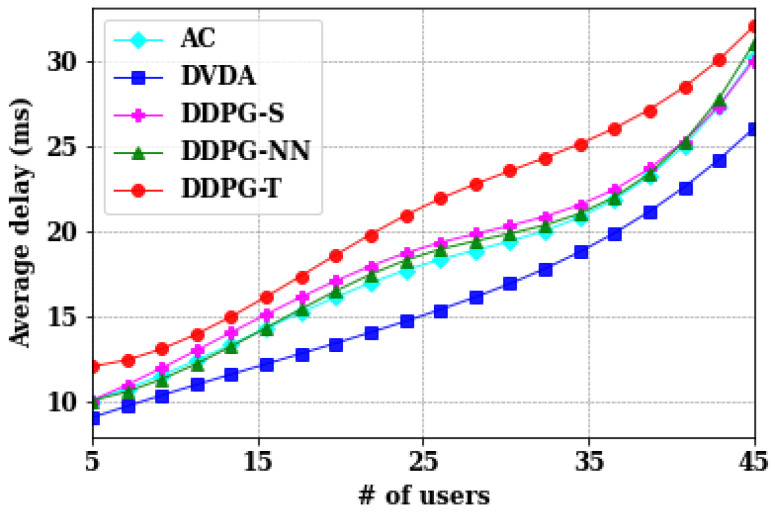
Comparison of different algorithms on the average delay of users.

In real-world scenarios such as emergency response systems, the number of users (emergency responders) can be substantial. For our evaluation, we varied the number of users to 5, 15, 25, 35, and 45 while maintaining default model parameters for all the algorithms, AC, DDPG-NN, DDPG-S, DDPG-T, DVDA. Figure 4 illustrates that the average delay for users increases as the number of users rises. This underscores the challenges posed by scaling up UAV-based emergency response systems. Notably, the proposed DVDA approach exhibits lower average delays compared to others. Specifically, with 45 users, DVDA achieves an average delay of 26 ms, whereas AC shows an average delay of 30.1 ms, representing a 14.01% increase with respect to DVDA. Examining the increase from 5 to 45 users, AC experiences a delay increase from 10.2 ms to 30.1 ms, a rise of 19.9 ms. In contrast, DVDA demonstrates a delay increase of 17.1 ms over the same user range, which is 14.07% lower than AC. Moreover, DDPG-S, DDPG-NN, and DDPG-T have average delays of 28.6 ms, 28 ms, and 32 ms, respectively, which are higher than that of DVDA by 10%, 7.69%, and 23.07%, respectively.

#### 5.3.3. Impact on Average Transmission Time of LUAVs

**Figure 5:**
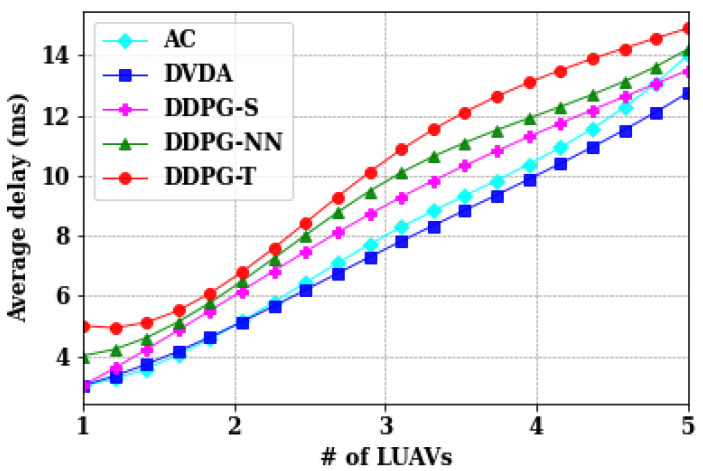
Comparison of different algorithms on average transmission time of LUAVs.

Depending on the nature of the emergency, the number of LUAVs may increase to support more MUAVs. However, as LUAVs increase, the average transmission time also increases due to the limited bandwidth capacity at the GBS. With more LUAVs, each receives less bandwidth, leading to decreased video transfer rates and increased transmission delays. In the evaluation, LUAVs are varied from one to five while keeping other network parameters constant. Figure 5 illustrates the impact of LUAV numbers on average transmission time. Notably, DVDA algorithm demonstrates reduced transmission time as LUAVs increase from three to five in comparison to AC, DDPG-S, DDPG-NN, and DDPG-T. Conversely, both AC and DVDA exhibit similar performance when LUAVs are less than or equal to two.

#### 5.3.4. Impact of Fairness on Users and LUAVs

**Figure 6:**
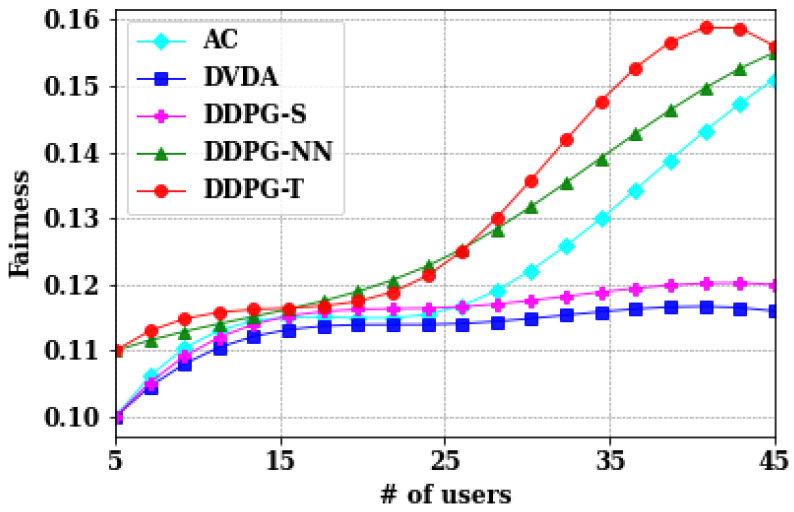
Comparison of different algorithms on average transmission time of users.

**Figure 7:**
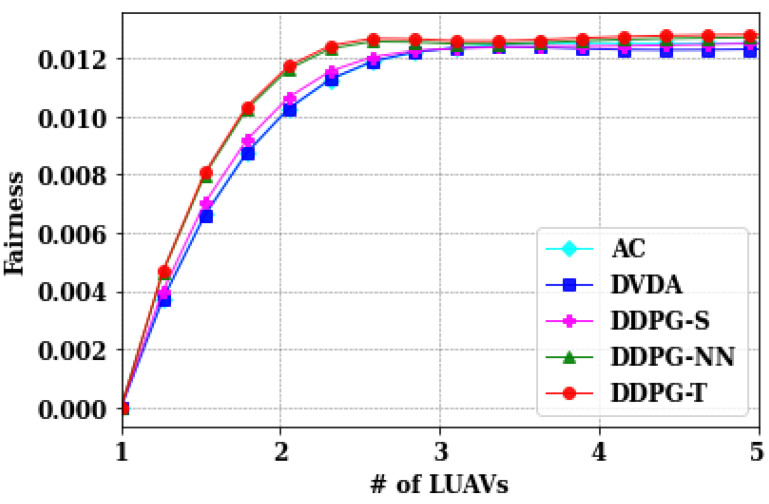
Comparison of different algorithms on average transmission time of LUAVs.

As described in Section 2.4, the fairness among users and LUAVs largely signifies the standard deviation of the total delay among users and transmission delays among the LUAVs. The main aim was to minimize the fairness such that the discrepancy of video data received at the user end can be minimized. Considering the same number of users, 5, 15, 25, 35, and 45, the fairness is evaluated among users as shown in Figure 6, as well as the fairness among LUAVs as shown in Figure 7.

As observed in Figure 6, the fairness among users increased as the standard deviation among users increased when the number of users increased from 25 to 45. This increase in standard deviation is primarily attributed to the individual increase in total delay, which escalates with the system’s complexity as the number of users rises.

Similarly, for LUAVs, the fairness increased due to an increase in standard deviations in the transmission time. Although the performance of all the models is in very close proximity for LUAVs, DVDA still showed slightly better performance.

#### 5.3.5. Impact of Video Data Size on Average Delay for Users

**Figure 8:**
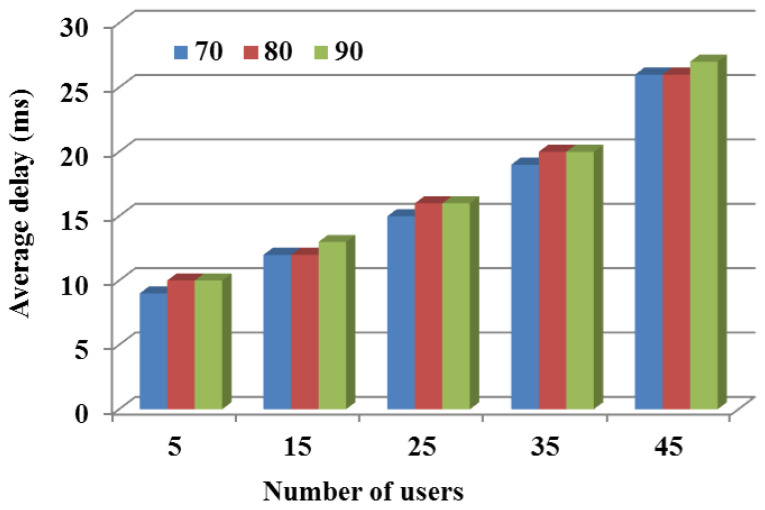
Comparison of different algorithms on the QoE of the system.

In order to visualize the effect of video data size on the average delay of users, using DVDA, a different set of the data size values was used: 70 MB, 80 MB, and 90 MB, and the number of users were varied to 5, 15, 25, 35, and 45. Throughout this analysis, all other parameters were kept constant. The results in Figure 8 shows that with an increase in video data size, the average delay for users is increased without a significant rise in the standard deviation among them. For instance, when the number of users was 5 and the data size was 70 MB, 80 MB, and 90 MB, the average delay was 9 ms, 10 ms, and 10 ms, respectively, indicating no significant increase in delay. This demonstrates that our DVDA algorithm adaptively distributed bandwidth and video trans-rating among the users and LUAVs.

#### 5.3.6. Impact of Video Data Size on System

**Figure 9:**
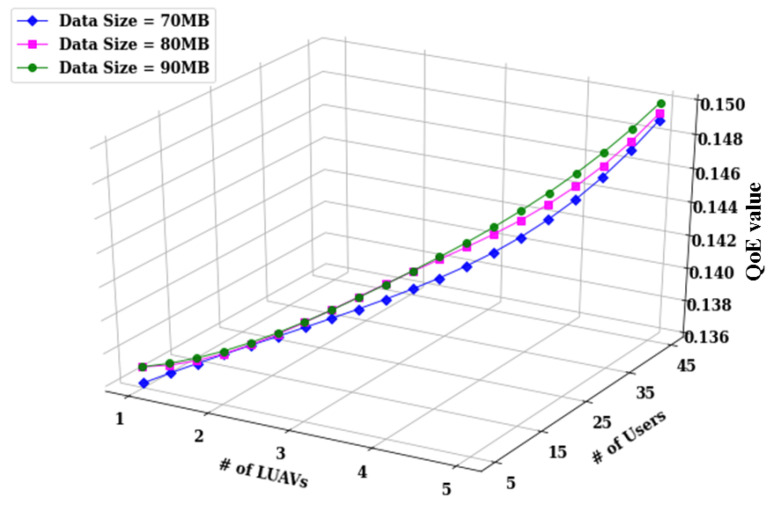
Comparison of different video data size on the QoE of the system.

The performance of the overall UAV-based video streaming system in the proposed DVDA algorithm is assessed based on QoE, defined by Equation (32). Video data sizes of 70 MB, 80 MB, and 90 MB are evaluated under varying conditions of users numbers 5, 15, 25, 35, and 45 and number of LUAVs 1, 2, 3, 4, and 5, while other parameters remain constant. The consolidated outcomes in Figure 9 underscore effectiveness of DVDA in minimizing the overall system QoE as the user count, LUAVs, and video data size increase. Specifically, the average QoE values of 0.136, 0.137, and 0.137 are observed for video data sizes of 70 MB, 80 MB, and 90 MB, respectively, with five users and one LUAV. When the user count increases to 45, the average QoE values are higher at 0.149, 0.1494, and 0.150 maintaining the same video data sizes. The marginal standard deviation of 0.0004 across these measurements indicates minimal variation under dynamic system conditions. Furthermore, the overlapping results illustrate the adaptive capability of DVDA in minimizing the system QoE. The weighting factors $\lambda_{1}$ and $\lambda_{2}$ are set to be 0.6 and 0.4, respectively, based on their performance as shown in Figure 3.

#### 5.3.7. Impact on Average System Reward

**Figure 10:**
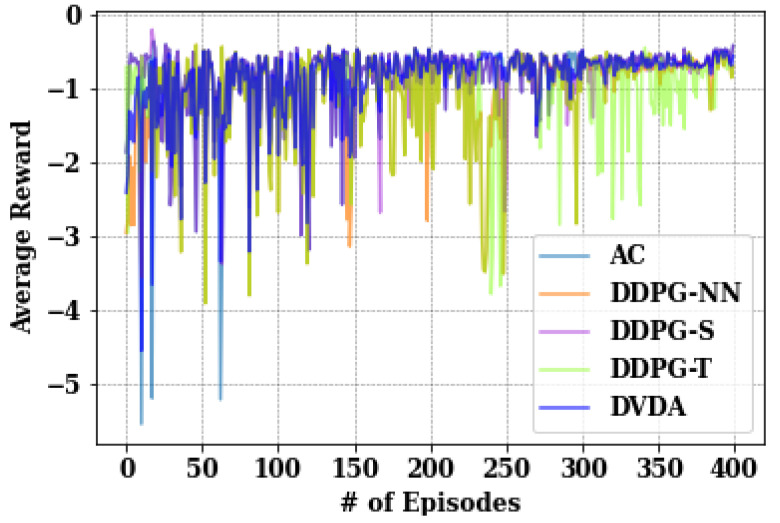
Comparison of different policies on the average reward of the system.

The performance of the proposed DVDA algorithm against AC, DDPG-S, DDPG-NN, and DDPG-T, using identical hyperparameters across all models. As depicted in Figure 10, DVDA, AC, and DDPG-S exhibited relatively smoother reward curves compared to DDPG-NN and DDPG-T, indicating challenges in convergence. Furthermore, AC, DVDA, and DDPG-S achieved lower total average scores of −347.468, −329.516, and −358.904, respectively, while DDPG-NN, DDPG-T obtained the highest total reward scores of −399.4 and −432.667, respectively. In the current analysis, a lower reward is preferable, as the primary aim is to minimize the system QoE, targeting reduced total user delay, LUAV transmission time, and transmission time standard deviation among users. Therefore, negative rewards were used to align with this objective, where a lower average reward signifies better model performance. Throughout the experiment, we maintained $\lambda_{1}=0.6$ and $\lambda_{2}=0.4$, with 1 MUAV, 1 LUAV, and 5 users for all models.

### 5.4. Limitations and Future Work

While our study offers valuable insights into single-agent-based bandwidth allocation systems, it is essential to acknowledge certain limitations that warrant further exploration. Presently, our framework operates within the constraints of a single-agent paradigm, focusing predominantly on optimizing bandwidth allocation alongside video trans-ration and trajectory control. However, the potential efficacy of bandwidth allocation systems can be significantly enhanced by transitioning towards a distributed approach, capitalizing on the capabilities of multi-agent scenarios. By embracing a distributed architecture, we unlock a multitude of potential improvements in bandwidth allocation, video trans-ration, and trajectory control, where multiple agents collaborate and coordinate actions.

In multi-agent scenarios, challenges such as interference and spectrum sharing become more pronounced. For instance, multiple MUAVs or LUAVs or base stations covering the same area may introduce significant interference, which needs to be accounted for as described in Equation (13). This additional complexity makes the DRL modeling more intricate, as it requires precise coordination and communication between agents to mitigate interference effectively. Thus, the additional interference parameter in Equation (13) may provide useful solutions in the accurate modeling of the real-world conditions and mitigating the effects of signal interference. However, the interference parameter in Equation (13) is ignored in the current analysis due to the single-agent approach.

Additionally, the current study primarily focuses on the GBS being the central agent. However, exploring collaborative efforts between LUAVs and the GBS, where both the LUAVs and GBS can act as collaborating agents, presents a promising avenue for optimization. LUAVs can significantly expedite direct video transmission to users, potentially reducing the overall transmission time. Moreover, delving deeper into LUAVs for dynamic bandwidth allocation among MUAVs could yield more tailored and efficient allocation strategies. Furthermore, expanding trajectory control beyond LUAV velocity to encompass flight angle, battery constraints, and power consumption for both MUAVs and LUAVs could further enhance bandwidth allocation, video transcoding, and trajectory control in emergency response systems.

The incorporation of edge–fog–cloud computing [26] or cloud–edge–meta operating systems [27] stands to enhance the emergency response system by optimizing spectrum and computing resource utilization. Furthermore, leveraging 5G technology [28] promises significant improvements in data transmission speeds and latency reduction, thereby bolstering the overall system performance. Future work will involve developing and integrating these multi-agent systems and interference parameters into the existing framework.

## 6. Conclusions

This study has explored the complex challenges and opportunities presented by integrating UAVs with edge servers and end users to establish an efficient emergency response system focusing on seamless video data transmission. The critical issue of live video streaming latency is addressed, particularly important during emergencies where timely information dissemination is essential. By harnessing edge computing and considering the mobile nature of UAVs, an adaptive resource management strategy is proposed to optimize system performance within the constraints of limited UAV resources. The proposed DVDA incorporates the average total delay for users, average transmission delay for LUAVs, the fairness factor between users and LUAVs, and trajectory control to formulate an efficient bandwidth allocation scheme and video trans-rates scheme among users and LUAVs. Moving forward, future research should focus on addressing the limitations identified in this study to develop more sophisticated video transmission systems tailored specifically for emergency response scenarios. This includes exploring advanced techniques and technologies to further enhance system efficiency and effectiveness during critical situations.

## Figures and Tables

**Table 1:** Summary of notations related to the system model.

Notation	Description
*t*	Time slot length calculated as t=T/N
U	Set of collaborative users, where each user is denoted by *u*
G	Set of GBS, where each GBS is denoted by *g*
M	Set of MUAVs, where each MUAV is denoted by *m*
L	Set of LUAVs, where each LUAV is denoted by *l*
$p_{t}^{u}$	Position of each user defined by $\left(x_{t}^{u},y_{t}^{u},0\right)$
$p_{t}^{g}$	Position of each GBS defined by $\left(x_{t}^{g},y_{t}^{g},0\right)$
$p_{t}^{l}$	Position of each LUAVs defined by $\left(x_{t}^{l},y_{t}^{l},h_{t}^{l}\right)$
$p_{t}^{m}$	Position of each MUAV is denoted by $\left(x_{t}^{m},y_{t}^{m},h_{t}^{m}\right)$
$v_{t}^{u}$	Velocity of each user *u*
$v_{t}^{l}$	Velocity of each user *l*
$v_{t}^{m}$	Velocity of each user *m*
ϱ	Damping factor
$d_{t}^{g,u}$	Distance between user *u* and GBS *g*
$d_{t}^{l,g}$	Distance between LUAV *l* and GBS *g*
$d_{t}^{m,l}$	Distance between MUAV *l* and LUAV *l*
$\Phi_{1}$, $\Phi_{2}$, $\Phi_{3}$	Channel gains between user to GBS, LUAV to GBS and MUAV to LUAV
$f_{t}^{g,u}$	Channel fading between user *u* and GBS *g*
$\alpha_{1}$,$\alpha_{2}$	Atmospheric attenuation coefficient
$B_{t}^{g}$	Total bandwidth available to GBS at any time slot *t*
$B_{t}^{u}$	Total bandwidth allocated to users by GBS at any time slot *t*
$B_{t}^{l}$	Total bandwidth allocated to users by GBS at any time slot *t*
$\Psi_{t}^{g,u}$	Video transfer rate between GBS, *g* and user *u* at any time slot *t*
$\Psi_{t}^{l,g}$	Video transfer rate between LUAV *l* and GBS *g* at any time slot *t*
$\Psi_{t}^{m,l}$	Video transfer rate between MUAV m and LUAV *l* at any time slot *t*
$\rho_{t}^{u,g}$	Transmit power between user *u* and GBS *g* at any time slot *t*
$\rho_{t}^{l,g}$	Transmit power between LUAV *l* and GBS *g* at any time slot *t*
$\rho_{t}^{m,l}$	Transmit power between MUAV *m* and LUAV *l* at any time slot *t*
$\varpi_{t}^{m,l}$	Interference parameter between MUAV and LUAV at any time slot *t*
$\chi_{1}$,$\chi_{2}$,$\chi_{3}$	Additive Gaussian noise
$T_{t}^{u,g}$	Transmission delay between user *u* and GBS *g* at any time slot *t*
$T_{t}^{m,l}$	Transmission delay from MUAV *m* to LUAV GBS *l* at any time slot *t*
$T_{t}^{l,g}$	Transmission delay from LUAV *l* and GBS *g* at any time slot *t*
$T_{t}^{g,u}$	Transmission delay from user *u* to GBS *g* at any time slot *t*
$T_{t,C}^{l}$	Processing delay at LUAV *l* at any time slot *t*
$T_{t,Q}^{l}$	Processing delay at LUAV *l* at any time slot *t*
$T_{t,E}^{l}$	Transferring delay at LUAV *l* at any time slot *t*
$\Phi_{t}^{l,g}$	Video trans-rated ratio between LUAV *l* and GBS *g* at any time slot *t*
$\Phi_{t}^{g,u}$	Video trans-rated ratio between GBS *g* and user *u* at any time slot *t*
$D_{t}^{k}$	The original video data size for $k^{th}$ video sequence at any time slot *t*
$q_{t}^{m}$	The CPU related parameters of the MUAV *m* at any time slot *t*
$\xi_{t}^{u,g}$	Priority of user *u* requesting video data at GBS *g* at any time slot *t*
$\xi_{t}^{l,g}$	Priority of LUAV *l* requesting video data at GBS *g* at any time slot *t*
$T_{t,C}^{g,u}$	Processing delay at GBS *g* for user *u* at any time slot *t*
$T_{t,Q}^{g,u}$	Queuing delay at GBS *g* for user *u* at any time slot *t*
$T_{t,E}^{g,u}$	Transferring delay at GBS *g* for user *u* at any time slot *t*
δ	Maximum deadline of the task
$F_{1}\left(U\right)$	User’s fairness
$F_{2}\left(L\right)$	LUAV’s fairness
${⅁}_{t}$	QoE of the system at time slot *t*

## Data Availability

No new data were created or analyzed in this study.
